# Getting sweeter: new evidence for glucose transporters in specific cell types of the airway?

**DOI:** 10.1152/ajpcell.00140.2022

**Published:** 2022-11-21

**Authors:** Deborah L. Baines, Stanislavs Vasiljevs, Kameljit K. Kalsi

**Affiliations:** Institute for Infection and Immunity, St George’s, University of London, London, United Kingdom

**Keywords:** airway, epithelium, glucose, glucose transporter, single-cell RNA sequencing

## Abstract

New technologies such as single-cell RNA sequencing (scRNAseq) has enabled identification of the mRNA transcripts expressed by individual cells. This review provides insight from recent scRNAseq studies on the expression of glucose transporters in the epithelial cells of the airway epithelium from trachea to alveolus. The number of studies analyzed was limited, not all reported the full range of glucose transporters and there were differences between cells freshly isolated from the airways and those grown in vitro. Furthermore, glucose transporter mRNA transcripts were expressed at lower levels than other epithelial marker genes. Nevertheless, these studies highlighted that there were differences in cellular expression of glucose transporters. GLUT1 was the most abundant of the broadly expressed transporters that included GLUT8, 10, and 13. GLUT9 transcripts were more common in basal cells and GLUT12 in ionocytes/ciliated cells. In addition to alveolar cells, SGLT1 transcripts were present in secretory cells. GLUT3 mRNA transcripts were expressed in a cell cluster that expressed monocarboxylate (MCT2) transporters. Such distributions likely underlie cell-specific metabolic requirements to support proliferation, ion transport, mucous secretion, environment sensing, and airway glucose homeostasis. These studies have also highlighted the role of glucose transporters in the movement of dehydroascorbic acid/vitamin C/myoinositol/urate, which are factors important to the innate immune properties of the airways. Discrepancies remain between detection of mRNAs, protein, and function of glucose transporters in the lungs. However, collation of the data from further scRNAseq studies may provide a better consensus and understanding, supported by qPCR, immunohistochemistry, and functional experiments.

## Graphical abstract

**Figure:**
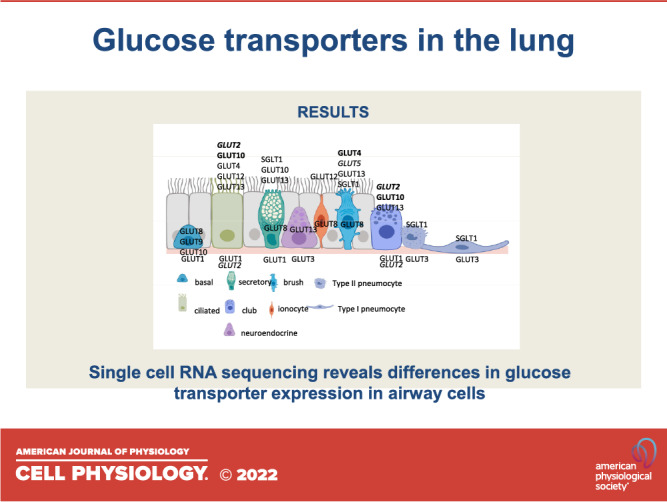

## INTRODUCTION

The recent development of new technologies such as single-cell RNA sequencing (scRNAseq) has enabled the identification of the mRNA transcripts expressed by single cells of many tissues including the airway. This review considers this exciting new technology and the information provided by several recent studies with a view to shedding further light on the cellular expression of mRNAs of glucose transporters and their functions in the lung.

### Cells of the Lung

**Figure 1.**
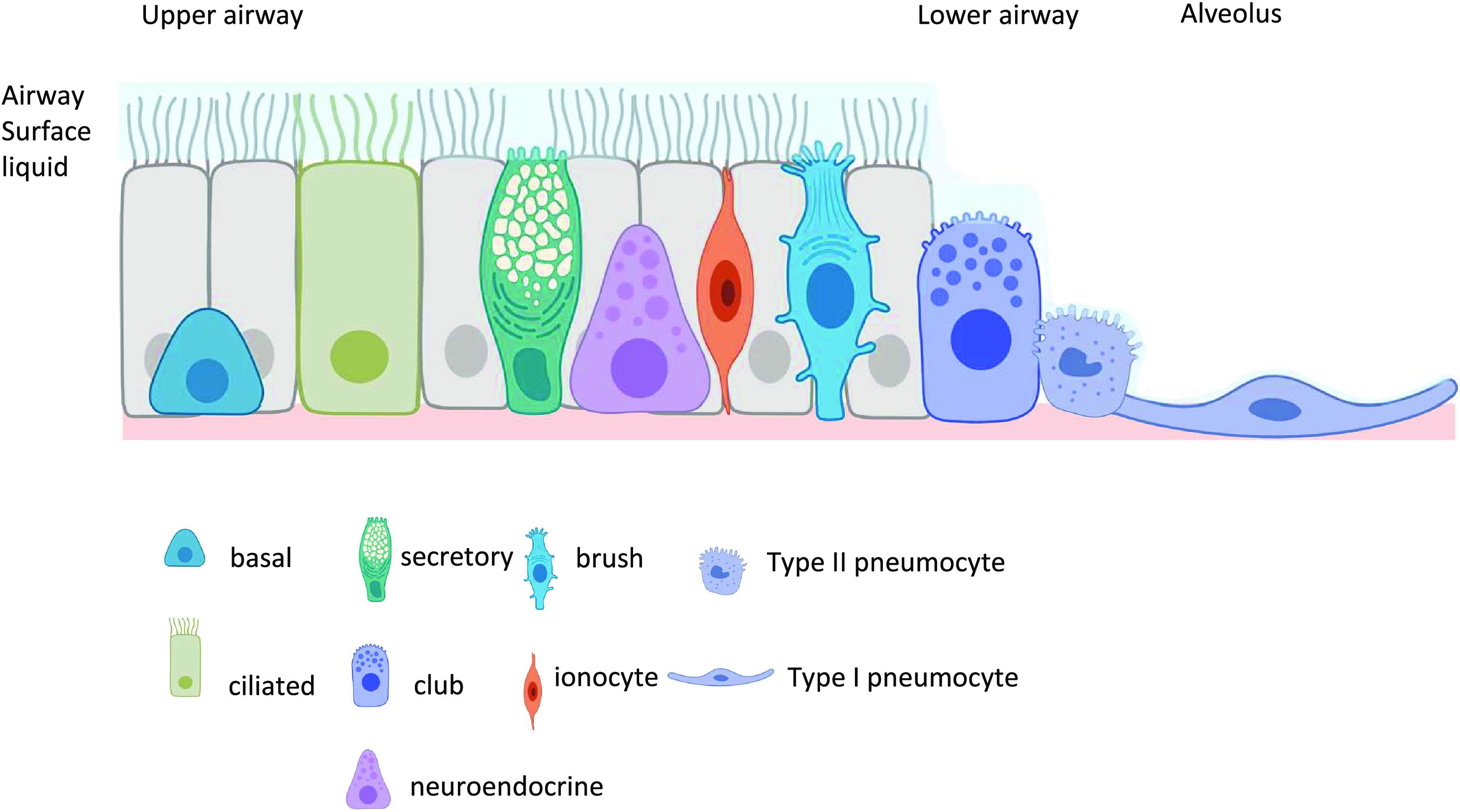
Schematic diagram of the key cells of the airway epithelium is shown with their approximate locations (upper airway, lower airway, and alveolus). The diagram does not indicate the relative contribution of individual cells to the airway epithelium or how these change from proximal to distal lung. The airway cells support a continuous layer of airway surface liquid (ASL) on the luminal surface which decreases in depth from the trachea to the alveoli.

The conducting airways of the lung are lined with a pseudostratified epithelium. The epithelium becomes columnar and cuboidal as it progresses from the bronchi toward the terminal bronchioles and becomes squamous interspersed with cuboidal cells in the alveoli (1; Fig. 1). The cells of the epithelium support a wide range of ion/solute transport mechanisms that regulate the volume and composition of a continuous layer of fluid (airway surface liquid; ASL) that is ∼7–10 µM in depth in the upper airways but reduces to 0.1 µM in the alveoli [so as not to impede gas exchange (2)]. ASL is critical for innate defense against infection (3). The upper airways predominantly consist of basal cells that are the key progenitor cells of the airway, goblet cells that secrete mucins into the ASL, and ciliated cells that move mucus and debris out of the airway (the mucociliary escalator), a process which is compromised in respiratory diseases such as cystic fibrosis (CF) (4–6). The epithelium also contains rarer cells such as tuft cells/brush cells, which support microvilli extending into the ASL and contain mRNAs and proteins associated with a sensory phenotype such as taste receptors (7) and pulmonary neuroendocrine cells (PNEC) that play a key role in airway immune function by secreting neuropeptides and bioactive amines (8). Mucus-secreting cells become fewer and club/clara exocrine cells become more frequent in the bronchiolar epithelium where they can serve as progenitors for ciliated cells. They secrete clara cell secretory protein (CCSP), otherwise known as uteroglobin, which has an important immunomodulatory function, lipids, and other protective components for the small airways (9). Finally, lining the alveoli, are alveolar type I and type II pneumocytes. Alveolar type II cells are small cuboidal cells with numerous microvilli on the luminal surface that secrete surfactant proteins and actively maintain a reduced ASL volume in the distal lung. They are considered progenitors for type I cells, which are estimated to cover 95% of the alveolar surface. These are thin cells with a large surface area and a continuous basement membrane that is fused with endothelial cells of the capillaries to enable efficient gas exchange (10; Fig. 1).

### Glucose Transport, Homeostasis, and Respiratory Disease

Glucose transport across lung epithelial cell membranes is mediated either by members of the *SLC2A* gene family of facilitative glucose transporters (GLUT) or by members of the *SLC5A* gene family of solute transporters that include sodium-coupled glucose transporters (SGLT). The facilitative glucose transporters (GLUT1-14) transport glucose and other substrates with differing affinity. Similarly, SGLT members 1, 2, and 4 transport glucose with differing affinity. SGLT3 is thought to act as a glucose sensor (Table 1).

**Table 1.** Facilitative and sodium coupled glucose transporters

Gene	Transporter	Substrate	K_m_
*SLC2A1*	GLUT1	Glucose	3 mM
*SLC2A2*	GLUT2	Glucose, galactose, manose, glucosamine	17 mM
*SLC2A3*	GLUT3	Glucose, galactose, manose, maltose, xylose, DHA	1.4 mM
*SLC2A4*	GLUT4	Glucose, glucosamine,DHA	5 mM
*SLC2A5*	GLUT5	Fructose	6 mM
*SLC2A6*	GLUT6	Glucose	>1 mM
*SLC2A7*	GLUT7	Glucose, fructose	0.3 mM (11)
*SLC2A8*	GLUT8	Glucose, fructose, galactose	0.3 mM
*SLC2A9*	GLUT9	Glucose, fructose, uric acid	0.6 mM (11)
*SLC2A10*	GLUT10	Glucose, galactose, DHA	0.3 mM (12)
*SLC2A11*	GLUT11	Glucose, fructose	0.16 mM
*SLC2A12*	GLUT12	Glucose, fructose, galactose	6.4 mM (13)
*SLC2A13*	GLUT13	Myoinositol	100 µM
*SLC2A14*	GLUT14	Glucose	ND (shares 95% homology GLUT3) (14)
*SLC5A1*	SGLT1	Glucose	0.4 mM
*SLC5A2*	SGLT2	Glucose	2.0 mM
*SLC5A4*	SGLT3	No glucose transport activity	Glucose sensor (15)
*SLC5A9*	SGLT4	Mannose, glucose	ND (15)

Gene name, transporter name, substrate transported, and K_m_ (glucose) values for transporters considered in this review. Further details on transporter characteristics can be found in Ref. 16. DHA, dehydroascorbic acid; GLUT, facilitative glucose transporter; SGLT, sodium-coupled glucose transporter.

Glucose transporters have a number of roles in the epithelial cells of the airway. Glucose uptake is required for cell metabolism to provide energy to drive cell proliferation and to support key functions such as ion transport and secretion of mucins and surfactant. However, glucose transporters also play an intrinsic role in maintaining glucose homeostasis across the airway epithelium and in maintaining the innate immune defense properties of the ASL (17).

**Figure 2.**
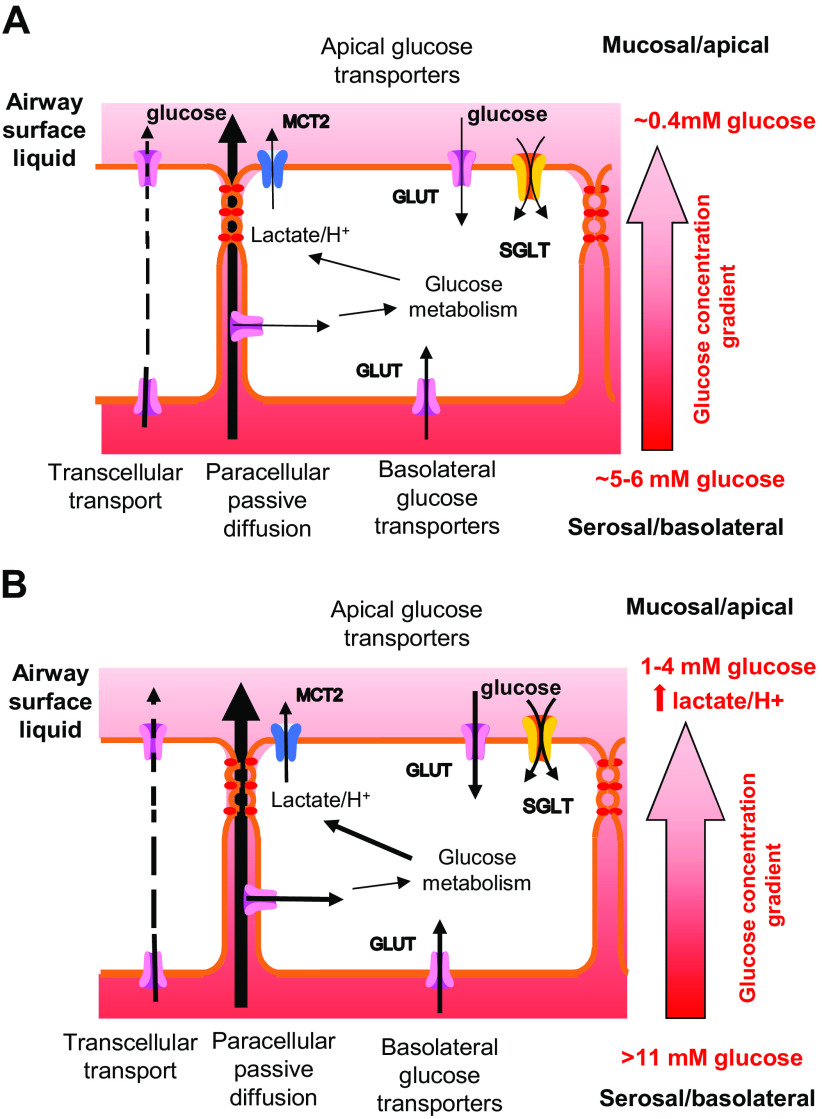
Airway glucose homeostasis *A*: in the healthy lung, airway surface liquid (ASL) glucose concentrations are maintained at around 0.4 mM (∼12 times lower than blood concentrations) by the restricted paracellular permeability to glucose of the tight junctions between airway epithelial cells and by glucose uptake into cells. This occurs through facilitative glucose transporters (GLUT) located on the mucosal/apical or serosal/basolateral surface of the airway where transport down localized concentration gradients is driven by intracellular glucose metabolism. Transcellular glucose transport can occur but is limited by cellular metabolism. In the distal lung, glucose uptake from the lumen is primarily via sodium-coupled glucose transporters (SGLT) driven by intracellular glucose and/or Na^+^ gradients. Glucose metabolism generates lactate which is transported out of the cell with H^+^ into the ASL via monocarboxylate transporter 2 (MCT2). *B*: airway glucose homeostasis is altered by hyperglycemia, which increases the gradient for glucose movement into ASL, and by airway inflammation, which alters tight junction protein expression and increases paracellular glucose leak. Glucose transport and metabolism are upregulated but ASL glucose concentrations also increase to up to 4 mM, particularly if hyperglycemia is associated with airway inflammation. Lactate/H^+^ secretion into the ASL also increases which can lead to acidification in diseases, such as cystic fibrosis where the neutralizing effect of ${\text{HCO}}_{3}^{-}$ secretion via CFTR is compromised. CFTR, cystic fibrosis transmembrane conductance regulator.

Glucose concentrations are ∼12.5 times lower in ASL (∼0.4 mM) than in plasma (5–6 mM) in humans and animals (18–20). In vitro, human bronchial epithelial cells (HBEC) can be grown on permeable supports at the air-liquid interface (ALI), to form a differentiated epithelial layer with different cell types (as shown in Fig. 1) that are linked by tight junctions. These epithelial layers exhibit distinct basolateral/serosal and apical/mucosal surfaces and produce ASL (21, 22) which, similar to in vivo, has a reduced glucose concentration of 0.3–0.6 mM when corresponding basolateral glucose concentrations are 5–10 mM (23, 24). The current model underpinning this phenomenon is that there is a gradient for passive paracellular diffusion of glucose into the ASL. Glucose accumulation in the ASL is restricted by the permeability of the tight junctions between the airway epithelial cells. Glucose transport from the serosal/basolateral compartment via facilitative glucose transporters (GLUTs) further limits transepithelial diffusion. Rapid metabolism of glucose maintains a low intracellular concentration of glucose, reducing the potential for transepithelial glucose transport and enabling the uptake of glucose from the mucosal/apical compartment (25). In the distal lung, the presence of sodium-coupled glucose transport (SGLT) in the luminal/apical membrane of the epithelial cells enables glucose to be transported against its concentration gradient with the potential to further reduce glucose concentrations in the alveolar lumen (26, 27; Fig. 2*A*).

Maintaining a low glucose concentration in the lung lumen is proposed to contribute to the innate immune defense properties of ASL. Elevation of blood glucose concentration (hyperglycemia, as seen in diabetes mellitus) and/or inflammation leads to increased glucose concentration in the ASL to ∼1–3 mM (19, 26, 28). Elevation of glucose in the ASL was shown to reduce the secretion of antimicrobial proteins in the sinonasal epithelium (29) and also leads to the formation of advanced glycation end products (AGEs) that have proinflammatory effects. Exposure of HBEC grown at air-liquid interface to increased basolateral glucose, increased glucose metabolism and lactate/H^+^ secretion into ASL via monocarboxylate transporters (MCT2). In bronchial epithelial cells from people with cystic fibrosis (CFBE), when basolateral glucose concentration was raised, the loss of the ability to secrete ${\text{HCO}}_{3}^{-}$ via the cystic fibrosis transmembrane conductance regulator (CFTR) led to a decrease in ASL pH, a factor known to contribute to the pathophysiology of CF disease (22; Fig. 2*B*).

Hyperglycemia is associated with the increased growth of respiratory pathogens, such as *Staphylococcus aureus* and *Pseudomonas aeruginosa* in people, in animal models and in vitro (26, 27, 30–32) by potentially providing additional growth substrates in the ASL, independent of effects on antimicrobial/immune factors (33). Viral infections have also been shown to be promoted by hyperglycemia. A recent study using artificial intelligence to analyze the published literature and generate computational models to investigate the relationship between diabetes and severe acute respiratory syndrome coronavirus 2 (SARS-CoV2) concluded that physiologically relevant ASL glucose concentrations of 1.2 mM promoted SARS-CoV-2 infection by reducing the activity of antimicrobial proteins, by glucose modification of surfactant protein which reduced its antiviral activity, and by facilitating angiotensin-converting enzyme 2 (ACE2) binding and increased endocytosis of the virus (34). Furthermore, changes in cellular glucose uptake and metabolism were also shown to drive viral replication (35, 36).

Glucose transporters play a key role in cancer progression by driving increased cellular metabolism and proliferation. It has been suggested that in rapidly proliferating tissues, glycolysis is the preferred mechanism of energy generation (37). As glycolysis produces less energy per glucose molecule than mitochondrial respiration, there is a requirement to increase glucose uptake to meet cellular demand. Thus, glucose transporters are upregulated in a wide range of lung cancers and are targets for therapy (37–40). Interestingly, the types of glucose transporter elevated in specific cancers may not necessarily reflect those thought to be present in the originator cell. Squamous cell cancers (derived from basal cells) frequently show elevation of GLUT1 but adenocarcinomas (which potentially arise from type II alveolar cells/club cells) were shown to elevate GLUT5 a fructose transporter and SGLT2, a transporter normally restricted to the kidney and for which there is little evidence in the normal lung (26, 37).

The role of glucose transporters in the transport of other substrates, such as fructose, myoinositol, urate, and dehydroascorbate in the lung is less well understood (41–44).

The identity of the key glucose transporters in the human lung has not changed significantly over the last few years (as reviewed in Ref. 45). Previously, studies indicated that GLUT genes were predominantly expressed in the adult trachea, bronchi, and bronchioles with SGLT1 in the distal lung. The most abundant mRNA transcripts identified encoded GLUT 1, 3, 8, 10, and 12. In human airway epithelial cells in culture GLUTs 1, 3, 4, 5, 6, 8, 9, 10, 12, and SGLT1, mRNAs were detected by quantitative polymerase chain reaction (qPCR) (46). But these studies did not define the cell types in which the transporters were expressed. Immunohistochemistry has helped to localize transporters but is hampered by the lack of good antibodies and cellular function by the lack of specific pharmacological inhibitors. The advent of new technologies, such as single-cell RNA sequencing, has the potential to provide more detailed information on the cell-specific expression of glucose transporters in the lung.

### Single Cell RNA Sequencing

This new and rapidly expanding technique reverse transcribes mRNAs of an individual cell and amplifies the complementary DNAs using PCR. Second-generation sequencing is then used to obtain sequences that are then compared with known databases to identify the genes from which the transcripts arise. This technology is different from DNA microarray studies that generally identifies differences in mRNA expression from potentially mixed populations of cells that have been subject to different treatments or those from nondisease versus disease.

ScRNAseq generates huge datasets of short-read RNA sequences from many individual cells. Thus, appropriate processing methods are required. Raw scRNAseq expression counts are normally standardized within samples using scaling factors. Reads per kilobase per million mapped (RPKM) (47) and fragments per kilobase of exon per million mapped (FPKM) were developed for single-end and paired-end RNAseq, respectively. To compare between samples, transcripts per kilobase million (TPM) is more commonly used. In this method, the sum of all TPMs in each sample is the same, enabling the proportion of reads or relative abundance of specific RNAs in each sample to be compared. Counts per kilobase million (CPM) is similar but uses depth normalized counts whereas TPM is length-normalized (and then normalized by the length-normalized values of the other genes) (48).

Using these outputs, scRNAseq data can then be further analyzed to provide information about the mRNA landscape within the cell and how it changes with disease. Cells with particular RNA expression profiles can be grouped into clusters that are associated with different cell types, to track the developmental trajectories of cell lineages and identify distinct cell populations in tissues such as the lung.

### Single Cell RNA Sequencing and Cells of the Lung

ScRNAseq data for the human airway has been reported from human bronchial epithelial cells (HBEC) and human tracheal epithelial cells (HTEC), which have been isolated from the airway and then grown ex vivo at air-liquid interface (ALI) into a differentiated pseudostratified bronchial epithelium containing different cell types. Data have also been obtained from cells directly isolated from different regions of the human lung. Studies have used mouse lung epithelial cells similarly cultured or freshly isolated (49–54; Table 2). The use of scRNAseq on these cells and tissues has confirmed the identification of cell clusters that express similar transcripts and are associated with the different types of airway epithelial cells described above. However, studies also demonstrated that while cells can be grouped according to these cell types, there is significant heterogeneity reflecting maturity, transitional cell states, and functional diversity (49). Furthermore, additional interrogation of the data led to confirmation of the presence of the ion channel-rich pulmonary ionocyte and a cluster of cells expressing mRNAs encoding the monocarboxylate transporter 2 (MCT2) (49), the function of which is not yet fully understood. The identification of these additional cell types and the additional functional heterogeneity in the airway epithelium have implications for the types of glucose transporters potentially expressed in individual cells. For example, the cells in which glucose transporters mediate the uptake of sugars for metabolism, those that regulate glucose homeostasis across the lung/airway epithelium, and those that transport molecules that serve less well-known purposes in the lung.

**Table 2.** Single cell RNA sequencing studies used in this review

Citation	Cell Source	Cell Types Identified	Data Type Available	Glucose Transporters Recorded
Deprez et al. (53)	Human lung cells obtained from 10 healthy volunteers from the nose to the 12th division of the airway tree biopsy/brushing including nasal, tracheal, intermediate bronchial, distal	Basal Cycling basal Suprabasal Secretory Ciliated Goblet PNEC Ionocyte ATI ATII	Log CPM	SLC2A1, 3, 4, 5, 6, 8, 9, 10, 11, 12 SLC5A1, 2, 9
Goldfarbmuren et al. (50)	Primary human tracheal cells from 15 healthy volunteers (HTEC) differentiated at an air-liquid interface (ALI)	Basal proliferating Basal proteasomal Basal differentiating Mucus secretory CiliatedKRT8^high^ PNEC Ionocyte/ Tuft SMG basal SMG secretory	Log CPM	SLC2A1, 3, 6, 10, 12 SLC5A1, 2
Montoro et al. (52)	Tracheal epithelial cells from 4 C57BL/6 wild-type mice and 2 *Foxj1*-EGFP ciliated cell reporter mice	Basal Ciliated ClubIonocyte PNEC Tuft	Log TPM + 1 Enriched genes with Log2 fold change	SLC2A12 SLC5A8
Plasschaert et al. (49)	Primary human bronchial epithelial cells (HBEC) from 4 healthy donors (Lonza) differentiated at an air-liquid interface (ALI) C57/BL6 male and female mice	Basal Brush+PNEC CiliatedIonocyte Secretory SLC16A7+ FOXN4+	TPM	SLC2A1-12 SLC5A1, 2, 4, 9
Ravindra et al. (51)	Primary human bronchial epithelial cells (HBEC) (Lonza) differentiated at an air-liquid interface (ALI) C57BL/6JN mouse lung	Basal Ciliated Club GobletIonocyte PNEC Tuft	Number of cells /Relative expression %	SLC2A1, 3, 10, 11, 12, 13 SLC5A1, 3
Travaglini et al. (54)	Resected human histologically normal lung tissue from 3 donors including bronchi (proximal), bronchiole (medial), and alveolar (distal) regions	ATI ATII Basal Club Ciliated Goblet PNEC	Average expression/ % expression	SLC2A1, 2, 3, 4, 5, 6, 8, 9, 10, 11, 12, 13 SLC5A1, 2, 4, 9

ATI, alveolar type I; ATII, alveolar type II; FOXN4+, cells expressing the forkhead box/winged-helix transcription factor FOXN4 a marker of multiciliated cell differentiation; KRT8^high^, cells expressing high levels of Keratin 8 a marker of epithelial differentiation; SLC16A7+, cells expressing the moncaboxylate transporter MCT2; PNEC, pulmonary neuroendocrine cells; SMC, submucosal gland.

### Single Cell RNA Sequencing and Glucose Transporters

Much of the data from scRNAseq studies have been made publicly available that allows further interrogation of RNA sequences derived from specific genes. The number of cells in a cluster in which RNA transcripts are detected (frequency, normally expressed as %) and the number of RNA transcripts recorded per cell can be obtained. Thus, scRNAseq has the potential to provide more detailed insight into the comparative expression of a broader range of glucose transporter RNA transcripts in specific cell types/clusters in the airway. This, in turn, can provide key clues to the function of glucose transporters in the lung.

Although the benefits of scRNAseq cannot be ignored, there are some limitations with scRNAseq data used for this purpose. The data only provide a single snapshot of RNAs expressed at a particular time in those experiments. Cell preparation including culture methodology and time in culture can affect the expression of RNAs. Processing, such as sequencing platforms and read depth (% of total transcripts sequenced) can vary significantly and data analysis often uses different normalization methodologies (see section *Single Cell RNA Sequencing*). This can limit comparison across studies. The sensitivity for low abundance transcripts is also limited and not all cellular genes are expressed at high levels (55). Furthermore, the abundance of mRNA transcripts in the cell may not necessarily reflect protein abundance or function, as the stability and translation of mRNAs into proteins are highly regulated. Nevertheless, with these caveats in mind, this review now considers the evidence for the cellular expression of mRNAs encoding glucose transporters of the lung.

### Single Cell Expression of GLUT mRNA Transcripts

**Figure 3.**
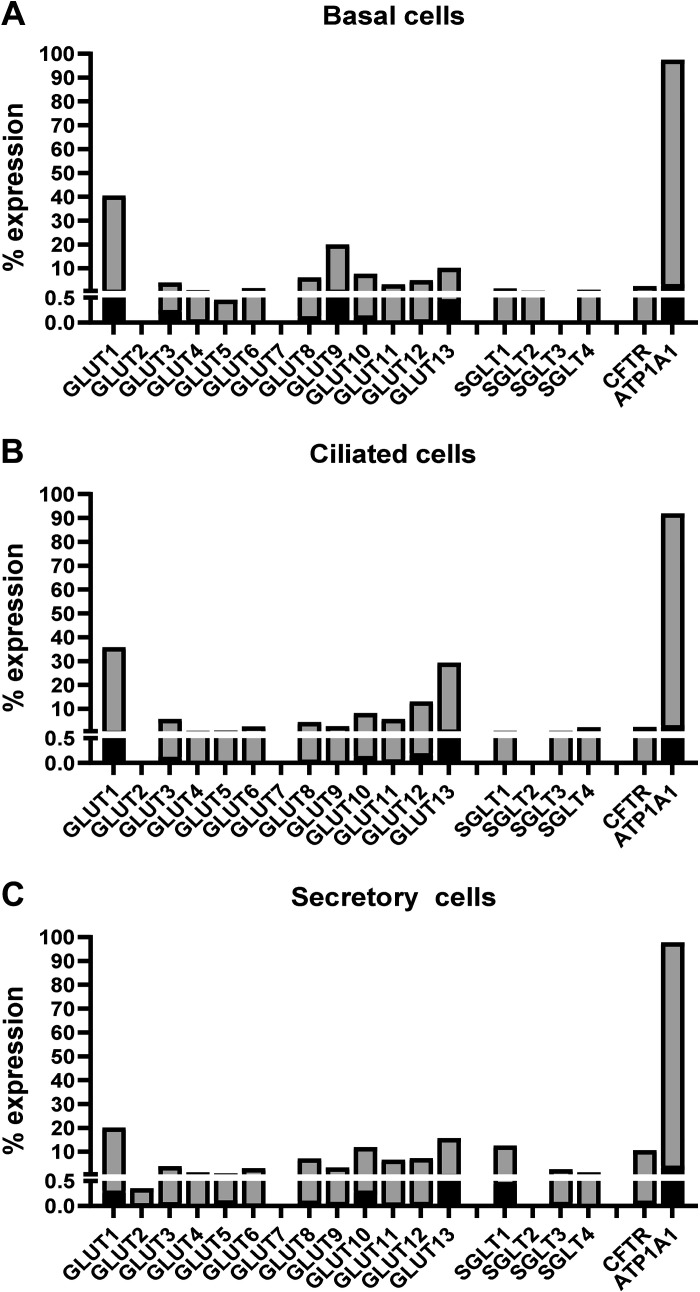
Mean % expression of glucose transporter mRNA transcripts (shown as transporter name GLUT1-13 and SGLT1, 2, 3, and 4) identified in basal (*A*), ciliated (*B*) and secretory cells of the airway (*C*) (light gray bars) and frequency of detection (*inset* black bar) analyzed from *n* = 2 studies where this data was available (Table 2). Expression of transcripts encoding the cystic transmembrane conductance regulator (CFTR, higher in secretory cells) and the Na^+^K^+^ATPase pump (*ATP1A1*, highly expressed in epithelial cells) are shown for comparative purposes. Differences in GLUT mRNA transcript expression profiles can be observed between these cell types. GLUT, facilitative glucose transporter.

Six studies that reported single-cell data for the human airway as outlined above were selected for further interrogation on the basis of the data availability, the lung cell types included, and the range of glucose transporters reported (even if not detected) (49–54; Table 1). The data in the studies varied in what transporters were recorded, the frequency and the abundance of transporters in different cell types, and whether cells were analyzed after in vivo collection or after culture in vitro. The data on type I and II cells was limited. It was notable that the relative expression of most glucose transporter mRNAs in these studies was generally low compared to epithelial markers such as the Na ^+^ K^+^ATPase pump (*ATP1A1*; Fig. 3, *A*, *B*, and *C*).

### Widely Expressed Glucose Transporters

**Figure 4.**
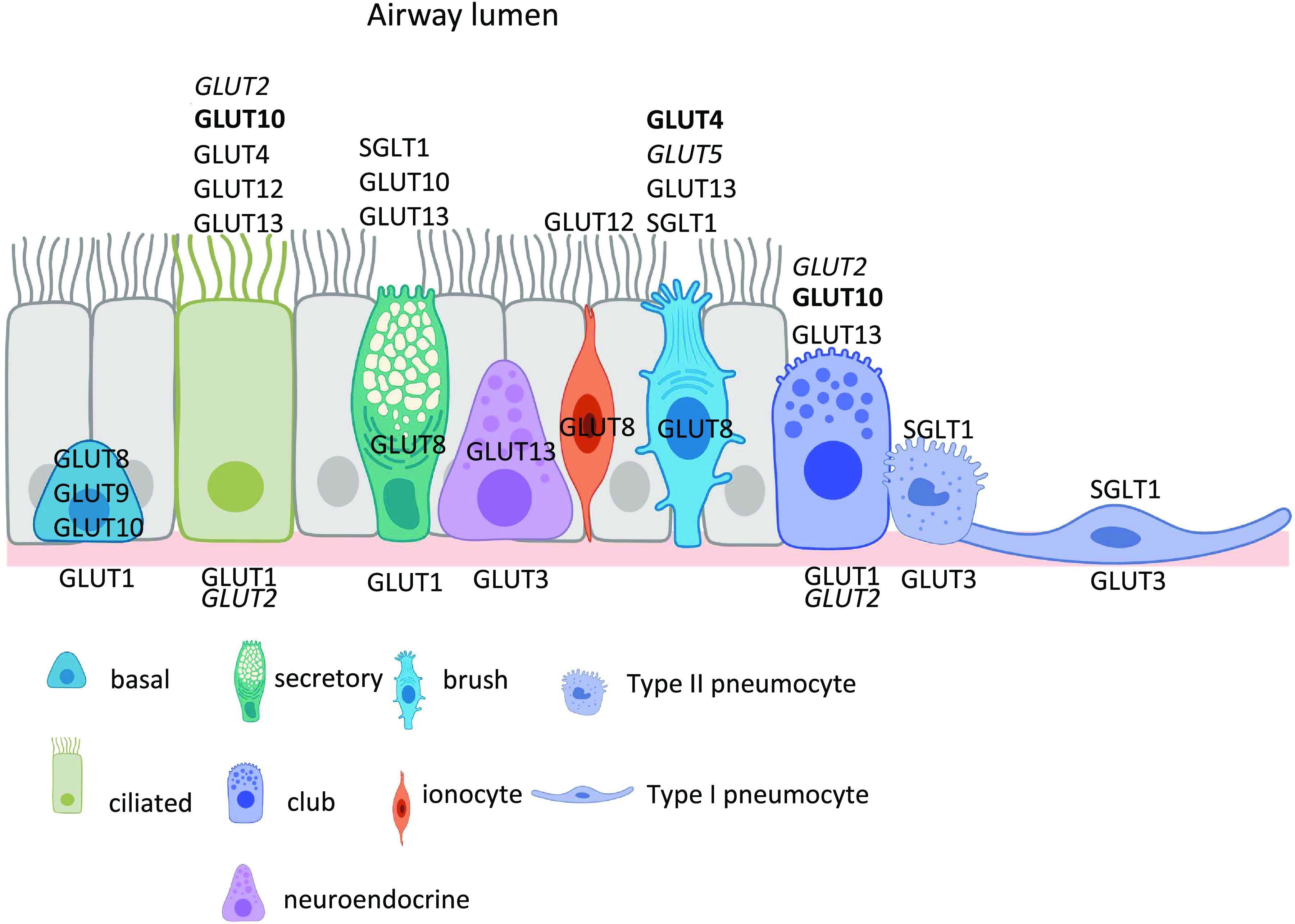
Glucose transporters associated with key cells of the airway epithelium are shown with their suggested localizations; luminal (above), cellular (within), and serosal (below). Standard type (mRNA only described), bold type (mRNA and protein described), italics (protein only described). The diagram does not indicate the relative contribution of individual cells to the airway epithelium or how these change from proximal to distal. It also omits the cell type associated with the expression of SLC17A6+ as further clarification of this phenotype in vivo is required. Created with BioRender.com.

Although there was variation between scRNAseq studies, generally GLUT1, 8, 10, and 13, transcripts were recorded in most airway epithelial cell types and were more abundant in basal cells (Fig. 3, *A*, *B*, and *C* and  Fig. 4).

### GLUT1

Four of the studies (three of which were from HBEC cultured at air-liquid interface) showed that GLUT1 is the most abundantly expressed transcript with higher frequency and transcripts in most cell types of the airway epithelium compared with other glucose transporters. The consensus was that GLUT1 mRNA transcripts were highest in the basal cells followed by ciliated and secretory cells supporting a role as a key mediator of glucose uptake to meet the potential energy demands of these cells (49–51, 53). Transcripts are less abundant in brush cells and ionocytes (53; Fig. 3, *A*, *B*, and *C* and  Fig. 4).

GLUT1 protein is primarily localized to the basolateral surface of primary HBEC differentiated at air-liquid interface but its presence in specific cell types was not further examined. Such a location would be consistent with the high levels of GLUT1 mRNA transcripts reported in basal cells and ciliated cells from cultured HBEC and support its proposed role in glucose homeostasis across the airway epithelium (23, 24). In murine lungs, GLUT1 is present in rat bronchial and primitive alveolar epithelial cells during the branching phase but becomes progressively less expressed from *wk 19* onward (56). The presence of GLUT1 has been shown to be important for the proliferation and maintaining the pool of airway progenitor cells (club cells). Lack of GLUT1 or glucose promotes differentiation to ciliated and goblet cells (57). This link with cell proliferation is supported by the finding that GLUT1 is upregulated in carcinomas of the lung and is associated with reduced cellular differentiation, increased tumor size, and metastasis (38, 58, 59). Thus, it is possible that GLUT1 protein is not highly expressed in vivo, especially in differentiated cells, but it is upregulated in vitro in response to culture conditions. This may explain why in human bronchial biopsies GLUT1 staining is associated with lymphocytes in the submucosa and not epithelial cells but is readily detected in HBEC in vitro.

### GLUT8

GLUT8 transcripts are present at low frequency in a range of epithelial cell types in vitro and are elevated in basal cells in one in vivo study (49, 53, 54). This is consistent with previous Northern blot data that showed very low mRNA abundance for GLUT8 in lung tissue (60). Little is known about the function of GLUT8 in lung epithelial cells, although it is also reported to play a role in early embryonic lung branching (34). It is present in other epithelial tissues, including the mammary gland, where it has a subcellular distribution yet contributes to deoxyglucose uptake (61). It has, therefore, been postulated to be an intracellular hexose transporter that localizes to lysosomal/endosomal membranes. Expression of GLUT8 correlates with high glucose and insulin levels, which potentially explains higher expression in cultured cells, and indicates a contribution to increased cellular metabolic capacity under these conditions (62). The generally ubiquitous nature of this transporter supports a role in the maintenance of epithelial glucose homeostasis (Fig. 3, *A*, *B*, and *C* and  Fig. 4).

### GLUT10

GLUT10 transcripts are present at low frequency in most epithelial cell types, although it is reported to be a marker of ciliated cells (together with GLUT1) and in secretory and goblet cells by others. Interestingly, GLUT10 mRNA was previously shown to be a highly abundant transcript in human airway tissues from trachea, bronchioles, and alveolar regions and in HBEC by northern blotting (12, 24, 63) (Fig. 3, *A*, *B*, and *C* and  Fig. 4).

**Figure 5.**
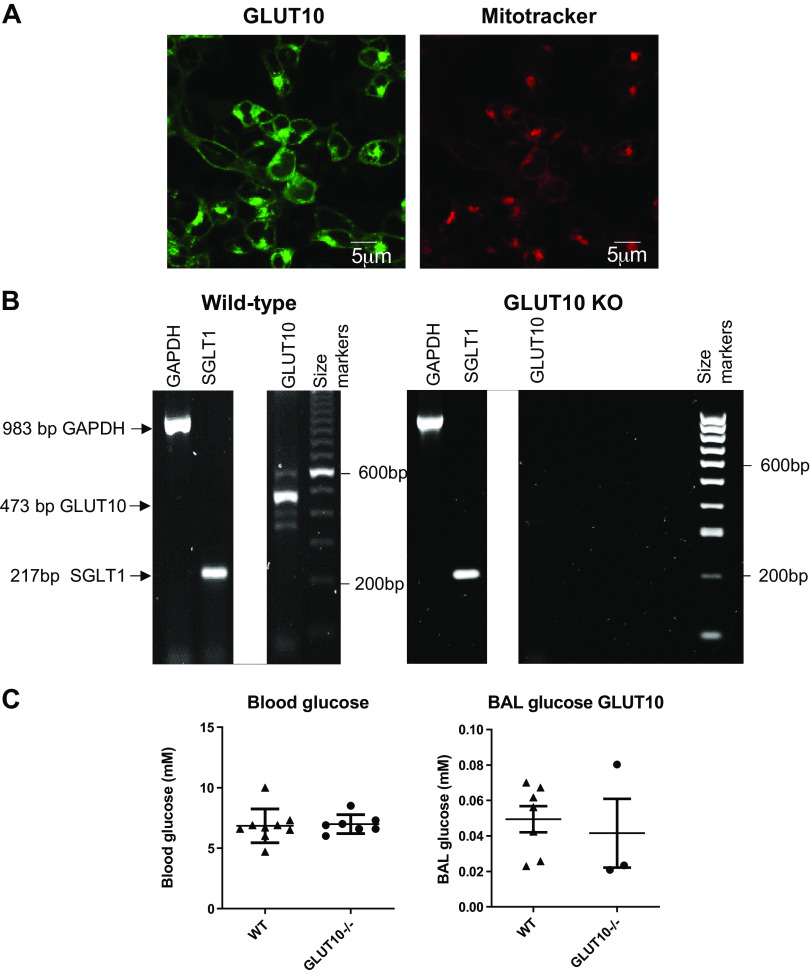
*A*: confocal microscope image of GLUT10 linked to GFP (GLUT10) expressed in HEK293T and the same cells stained with mitotracker dye to image mitochondria. GLUT10 is seen as green fluorescence and mitotracker deep red (Thermo Fisher, UK) as red fluorescence. Size markers are depicted at the bottom of each image. *B*: image of agarose gel showing polymerase chain reaction (PCR) products (white bands) corresponding to glyceraldehyde 3-phosphate dehydrogenase (GAPDH) (housekeeping gene), SGLT1 and GLUT10 obtained from lung tissue of male C3HeB/FeJ wild type (wild type) or GLUT10 deficient mice (GLUT10 KO). GAPDH and SGLT1 PCR products were present in both samples but GLUT10 was only present in wild type. Unrequired lanes between SGLT1 and GLUT10 have been digitally deleted from the image, the whole image can be provided upon request. *C*: blood glucose concentration and glucose concentration in bronchoalveolar lavage (BAL) of wild type and GLUT10 KO mice (using 1 mL total volume of sterile saline solution). Samples are shown as individual points with mean and standard deviation as shown. GLUT, facilitative glucose transporter; SGLT, sodium-coupled glucose transporter.

GLUT10 protein has been demonstrated in HBEC and the human H441 clara-like cell line consistent with the wide distribution of transcripts in airway cell types identified in vitro and in vivo (23, 24). GLUT10 localizes toward the apical domain of HBEC grown at air-liquid interface and expression of GLUT10 together with GLUT2 (see *Transcripts with Limited Detection in Airway Cells* for GLUT2) increases in H441 grown at the air-liquid interface after exposure to proinflammatory stimuli and this is associated with an increase in glucose uptake across the apical surface. GLUT10 has a high affinity for glucose (0.3 mM) thus, we and others proposed that GLUT10 may play a role in glucose uptake across the apical membrane of airway cells helping to maintain low glucose concentrations in the ASL (12). We have conducted a number of experiments to try and further elucidate the role of GLUT10 in the airway but it has not been easy. When expressed in HEK293T cells GLUT10- linked to green fluorescent protein (GFP) is localized to the membrane but also exhibited an intracellular distribution that correlates with a mitochondrial stain (Fig. 5*A*). GLUT2-linked to a red fluorophore (mcherry) or GLUT1-GFP were distinctly located to the cell membrane (64). Polymorphisms in GLUT10 are associated with arterial tortuosity syndrome (ATS). In vascular smooth muscle cells, GLUT10 is proposed to localize to mitochondria and/or endoplasmic reticulum membranes where it functions as a dehydroascorbic acid transporter and is associated with the compartmentalization of ascorbate within arterial cells (23, 65, 66). GLUT10 genetic variants associated with the disease were developed in C3HeB/FeJ mice to study the effects on the vascular system (67). We carried out preliminary investigations into the glucose concentration in the lungs of these mice compared with wild type at normoglycemia but found no difference (Fig. 5, *B* and *C*). Furthermore, when we cultured mouse tracheal epithelial cells (MTEC) from wild type and GLUT10-deficient mice at air-liquid interface, we found that, conversely, glucose uptake was increased across the apical membrane compared with wild type, but the glucose concentration in the ASL remained similar to that of wild type even when these cells were exposed to elevated basolateral glucose (unpublished data). Thus, it is possible that the inactivation of GLUT10 induces compensatory expression of another as yet unidentified transporter. The role of GLUT10 as a glucose transporter in airway epithelial cells, therefore, remains cloudy and its contribution to dehydroascorbic acid (DHA) uptake/compartmentalization in airway cells remains to be evaluated.

### GLUT13

Reporting of GLUT13 transcripts was heterogeneous. It is present at low levels in secretory, basal, and brush cells, although one study reports higher transcript levels with most enrichment in PNECs followed by the ciliated, club, and alveolar type II pneumoctes while another study reports enrichment in goblet cells. In vitro, GLUT13 is enriched in FOXN4+ cells, a marker of ciliated cell differentiation (49). There is little other documented evidence for GLUT13 (which transports myoinositol) in the lung. High expression levels of GLUT13 were reported in adenocarcinomas but not squamous cell carcinomas of the lung and this was associated with better overall survival (41). Myoinositol is important for the maturation of surfactant phospholipids and type II pneumocytes. In other tissues, inositol has been shown to downregulate the secretion of the proinflammatory cytokine IL-6 (68). Thus, GLUT13 could have an important role in maintaining the innate immune function of the ASL through the transport of myoinositol (Fig. 3, *A*, *B*, and *C* and  Fig. 4).

### Transporters Associated with Specific Cells

#### Basal cells.

GLUT9 transcripts were reportedly enriched in basal cells in one in vivo study and suprabasal cells in another (53, 54). It is known to play an important role in transporting urate in other tissues such as the placenta. Uric acid is detected in ASL and is considered to be a major low-molecular-mass antioxidant with the potential to scavenge reactive oxidant species (ROS) (43, 69). Thus, its potential role in transporting glucose and/or urate in basal cells requires further resolution (Fig. 3*B* and  Fig. 4).

#### SLC16A7 expressing cells.

GLUT3 transcripts were enriched in a cluster of cells expressing SLC16A7 (the monocarboxylate transporter MCT2) identified from HBEC cultured at air-liquid interface in vitro (49). GLUT3 was also enriched in alveolar type I and II cells in another in vivo study (54). Transcripts were detected at lower levels in basal and ciliated cells (49, 51, 53). The SLC16A7 cluster of cells is unusual in that a small proportion also expressed other GLUT transporter mRNA transcripts (GLUTs 4, 5, 6, 8, 9, 10, and 11) albeit at lower levels and not every cell expressed every transporter (Fig. 3, *A* and *B* and  Fig. 4).

Documented evidence for the presence of GLUT3 protein in the normal airway or airway epithelial cells and its role in the lung remains elusive (24, 70, 71). Nevertheless, it is highly expressed in lung tumor cells that have undergone epithelial to mesenchymal transition and some adenocarcinomas (38, 39, 59, 72). GLUT3 has a higher affinity for glucose (1.6 mM) and a fivefold greater transport capacity than GLUT1 which has led to the suggestion that GLUT3 is important for glucose uptake into cells when local glucose concentrations are low. Only inhibition of both GLUT1 and 3 in a mouse model of adenocarcinoma diminishes tumor development, supporting a role for GLUT3 in driving glucose metabolism (40). The association of GLUT3 with cells expressing MCT2 is of interest, although the association is not highlighted in vivo where MCT2 transporter mRNA transcripts were more broadly associated with PNEC, club, and basal cells (53, 54). We demonstrated that MCT2 transporters are present in the membrane (including the apical membrane) of HBEC and H441 cells grown at air-liquid interface (22). GLUT3 and MCT2 are expressed together in neurons where they have a role in glucose and lactate uptake respectively for energy generation. We showed that lactate is present in ASL of primary HBEC cultured at air-liquid interface in vitro. When these cells are exposed to basolateral hyperglycemia, lactate secretion via MCT2 transporters increases lactate concentration in the ASL (22). Although we cannot rule out that under certain conditions lactate could be taken up into the cell, we found no evidence that blockade of MCT1/2 transporters elevates lactate in ASL in normoglycemia or hyperglycemia, which would indicate a role for lactate uptake (22).

Taken together, the presence of such a wide range of glucose transporters in this cell cluster could indicate a metabolic role coupled with the secretion and uptake of lactate. This cluster is also reported to contain a large percentage of mitochondrial genes, which, as mitochondria play a critical role in detecting changes in intracellular homeostasis, could indicate cells under stress (49). This raises an important question as to whether this phenotype is associated with culture-induced stress of epithelial cells in vitro.

#### Ionocytes.

Data from some in vitro and in vivo studies indicate that GLUT12 transcripts are more frequently expressed and are more abundant in ionocytes followed by ciliated cells compared with other cell types (49, 50, 54; Fig. 4). Others identified GLUT12 as a marker of ciliated cells (52). GLUT12 protein is present in bovine lungs and rat lung bronchioles providing further evidence for a role in the airway (73, 74). In other tissues, such as the small intestine, GLUT12 is expressed in the apical/luminal membrane and high concentrations of glucose and/or insulin are reported to stimulate GLUT12 translocation to the brush border membrane (75). Insulin-sensitive glucose uptake has been documented in HBEC but was attributed to GLUT4 (46). Recent evidence shows that GLUT12 when reconstituted in proteolysosomes has almost double the affinity for glucose than GLUT1 (13). Glucose uptake by GLUT12 is inhibited by phloretin and cytochalasin B. It is also suggested that GLUT12 may transport dehydroascorbate in addition to sugars (13). Glucose normally moves through GLUTs by passive diffusion down a concentration gradient. But when GLUT12 is expressed in MDCK cells, it exhibits an ability to transport glucose against its concentration gradient when the pH is lowered from 7.4 to 5.0. This correlates with a decrease in intracellular pH which is inhibited by cytochalasin B indicating that active transport of glucose is coupled to H^+^ symport (76). When GLUT12 is expressed in *Xenopus laevis* oocytes, it transports more glucose in the presence of 100 mM extracellular Na^+^ than in Na^+^ free buffer. The addition of glucose also induces an inward current in the presence of Na^+^ which disappears when Na^+^ is removed. Taken together these data indicate that GLUT12 could act as an insulin-sensitive H^+^/Na^+^-glucose symporter (77). The ionocyte is a rare epithelial cell that supports high levels of ion channels and transporters such as the cystic fibrosis transmembrane regulator (CFTR), epithelial Na^+^ channel (ENaC), and the Na^ + ^K^+^ATPase pump (49). Energy (ATP) is required to drive ion channel function, the activity of CFTR is modulated by intracellular pH and CFTR can modify the pH of ASL via secretion of ${\text{HCO}}_{3}^{-}$. ENaC activity is regulated by both intracellular and extracellular Na^+^ concentration (78). If GLUT12 is indeed localized to ionocytes, it might be speculated that this transporter could have an important metabolic/sensory/regulatory role that co-ordinates with other channels and transporters to regulate ASL composition and function.

#### Secretory cells.

SGLT1 (SLC5A1) transcripts are considered to be expressed in the distal lung and not abundantly expressed in the airway. They are detected more than other members of the SLC5A family of transporters. An interesting finding was that scRNAseq data indicated that SGLT1 is located in secretory cells, with transcripts detected in goblet and mucous-secreting cells (49, 53, 54) (Fig. 2 and  Fig. 3*C*). SGLT1 protein has been demonstrated in the luminal membrane of cells in the distal lung but not airways or HBEC cultured at air-liquid interface in vitro (24, 27). However, SGLT1 was recently described in proximal lung organoids that were developed from human induced pluripotent stem cells (iPSCs) and which responded to prosecretory stimuli (79). This potentially fits with transcript hot spots in secretory cells (Fig. 3*A*, *B*, and *C* and  Fig. 4).

In the distal lung, transport via SGLT is driven by Na^+^ and glucose gradients. Cotransport of Na^+^ via SGLT1 contributes to fluid reabsorption and removal of glucose from the distal lung lumen but we and others have found no evidence for SGLT1 activity in HBEC grown at air-liquid interface (24, 26, 27, 80). In CF, loss of CFTR function and a consequent reduction in Cl^−^ transport results in compromised fluid secretion. In organoids from iPSCs from people with CF, SGLT1 protein was increased creating further potential for dehydration of the ASL, a critical pathophysiology leading to mucostasis, respiratory inflammation, and infection (79). The authors showed that inhibition of SGLT1 helped restore fluid secretion in these organoids, particularly when used in combination with CFTR modulators and suggested that SGLT1 could be a target for CF therapy. More work is now required to determine the role of SGLT1 in maintaining glucose and fluid homeostasis in the upper airway.

### Transcripts with Limited Detection in Airway Cells

There remain significant inconsistencies between mRNA and protein expression for GLUT2 in the airway. GLUT2 mRNA is reportedly absent or very low in freshly isolated airway cells, yet GLUT2 protein was found in the epithelial cells from human bronchial biopsies by ourselves and others and therefore proposed to have an important role in maintaining airway glucose homeostasis (42, 70, 81). GLUT2 protein is also reported in chemosensory cells (81, 82). ScRNAseq from HBEC grown in vitro, shows GLUT2 transcripts in a very small percentage of secretory cells from two studies (49, 54; Fig. 3*C*). Yet GLUT2 protein was not identified in differentiated primary airway cells in vitro, even though many cells stained positive for differentiation markers and likely contained secretory cells (24). It is possible that these differences relate to the specificity/sensitivity of tools used to measure mRNA and protein but it does raise questions about the value of mRNA data alone, the translation of mRNA into protein, and whether processing or culture of primary epithelial cells alters the expression of GLUT proteins. From the data that are available, it seems unlikely that the presence of GLUT2 in secretory cells is linked to energetic requirement. GLUT2 has a low affinity for glucose (17 mM) which, given that normal blood glucose concentrations are 5–6 mM and corresponding ASL glucose concentrations are ∼0.4 mM, suggests it would be a poor transporter of glucose compared to GLUT1. Alternatively, GLUT2 has been shown to mediate vitamin C uptake (42). The authors suggested that as GLUT2 plays an important role in dehydroascorbic acid (DHA) (the oxidized form of ascorbic acid/vitamin C) uptake from the gut lumen, it is possible that it performs a similar function in the lung (42). Vitamin C is a key antioxidant in the airway, with anti-inflammatory properties. Vitamin C also regulates nasal ciliary beat frequency (83). Thus, GLUT2 could play a role in regulating levels of vitamin C in the ASL and consequently ciliary beat frequency. Furthermore, together with GLUT10, GLUT2 may help compartmentalize cellular vitamin C and DHA.

There is also scant evidence for GLUT4 and GLUT5 transcripts in vivo although transcripts were detected in a few brush/PNEC/secretory cells in HBEC in vitro. GLUT4 protein was detected in the human trachea, bronchioles, and in differentiated HBEC in ciliated and non-ciliated cells (46). Insulin promoted translocation of GLUT4 to the apical domain of HBEC cultured in in vitro and upregulated deoxyglucose uptake (a glucose analog transported by GLUTs) but not all was inhibited by Cytochalasin B, perhaps supporting the presence of other insulin-sensitive transporters such as GLUT12 (see *Ionocytes*) (46). GLUT5 is a fructose transporter and the protein is detected in the luminal membrane of solitary chemosensing cells and secretory cells of the rat trachea, consistent with in vitro ssRNAseq data. GLUT5 is also detected in basal cells and in normal lung adjacent to, and in squamous cell carcinomas. GLUT5 is significantly upregulated in these tumors which is associated with poor patient survival (44, 84). Fructose transport and utilization are proposed to drive adenocarcinoma cell growth by stimulating fatty acid synthesis, inhibiting AMP-activated protein kinase (AMPK), and stimulating the target of rapamycin complex 1 (mTORC1) activity to promote tumor growth. Pharmacological inhibition of GLUT5 inhibited tumor growth in vitro highlighting a potential therapy for such tumors (84). However, circulating levels of fructose in vivo are normally much lower than that of glucose (less than 0.1 mM) and the K_m_ of GLUT5 for fructose is >10 mM. Thus, how fructose drives tumor development remains unclear.

From the scRNAseq data analyzed, the detection of SGLT2, 3, and 4 mRNA transcripts was at the limit of detection providing little evidence of their expression in normal epithelial cells of the airway. That SGLT2 is upregulated in adenocarcinomas of the lung (37) indicates that transcriptional induction of this transporter, classically thought of as limited to the renal proximal tubule, can be induced in the lung. SGLT3 and SGLT4 mRNA transcripts are present in lung tissues by qPCR and SGLT3 is associated with brush cells in the gut (15). Thus, their airway localization remains to be determined.

## SUMMARY

Understanding which glucose transporters are present in the lung, their cellular location, and how they change in respiratory disease will provide further insights into the function of airway cells and their contribution to cancer progression, the regulation of ASL glucose homeostasis, and innate defense against infection. The data available from the scRNAseq studies investigated was variable in the transcripts that were recorded, the levels of expression, and the cell types in which transcripts were expressed. There were differences between HBEC cells grown at air-liquid interface in vitro and cells freshly isolated from the airways suggesting culture-induced changes in transporter expression. There also remain discrepancies between the detection of mRNAs and protein for glucose transporters in the lungs. The collation of data from further studies may provide a better consensus, supported by qPCR, and immunohistochemistry. Nevertheless, these studies have highlighted that there were differences in cellular glucose transporter expression which may not only underlie differences in metabolism to support cellular roles such as proliferation, ion transport, mucous secretion, and environment sensing but also their contribution to airway glucose homeostasis. They have provided new targets for investigation (e.g., GLUT12) and have identified potential roles for glucose transporters in the production of lactate and the transport of dehydroascorbic acid (DHA)/vitamin C and myoinositol which are factors important to the innate immune properties of the ASL.

Targeting glucose transporters to modify lung cell metabolism and ASL homeostasis in respiratory disease is a possibility but it is complicated by the ubiquitous nature of some transporters and the pliable potential of other transporters to provide alternative pathways for glucose uptake. Thus, specific and selective approaches will be required. High potency GLUT 1–3 inhibitors are being developed to suppress glucose transport and metabolism to restrict tumor growth (85). High glucose was shown to induce ACE2 expression (a receptor for SARS-CoV2) via GLUT1 in bronchial submucosal cells in vitro (86) and blocking glycolysis prevented SARs CoV2 replication (87). Promoting SGLT1 activity by elevating intracellular cAMP with terbutaline reduced infection in the lungs of mice by increasing glucose uptake from the ASL (27). Conversely, inhibition of SGLT1 activity in CF was proposed as a way to aid restoration of fluid volume in the ASL although this could lead to elevation of glucose in the ASL with further consequences for respiratory disease (26). There are undoubtedly challenges ahead but a better understanding of glucose transport in the airway and how it changes in disease will be critical for effective therapeutic intervention.

## DATA AVAILABILITY

Studies shown in Table 2 provide details on data availability. Other data can be made available upon reasonable request.

## GRANTS

This work was supported by WellcomeTrust (Grant No: 088304/Z/09/Z), the Medical Research Council (Grant No: MR/K012770/1), and Medical Research Council Collaborative Awards in Science and Engineering (MRC CASE) studentship award with AstraZeneca, Gothenburg, Sweden and St. George’s University of London Enterprise Award.

## DISCLOSURES

No conflicts of interest, financial or otherwise, are declared by the authors. 

## AUTHOR CONTRIBUTIONS

S.V. and K.K.K. performed experiments; D.L.B. and K.K.K. analyzed data; D.L.B. interpreted results of experiments; D.L.B. prepared figures; D.L.B. drafted manuscript; D.L.B. edited and revised manuscript; D.L.B. approved final version of manuscript.
